# A bioinformatic survey of distribution, conservation, and probable functions of LuxR solo regulators in bacteria

**DOI:** 10.3389/fcimb.2015.00016

**Published:** 2015-02-24

**Authors:** Sujatha Subramoni, Diana Vanessa Florez Salcedo, Zulma R. Suarez-Moreno

**Affiliations:** ^1^Grupo de Bioprospección, Facultad de Ingeniería, Universidad de La Sabana, Campus del Puente del ComúnChía, Colombia; ^2^Instituto de Biotecnología, Universidad Nacional de Colombia, Ciudad UniversitariaBogotá, Colombia

**Keywords:** LuxR solos or orphans, quorum sensing, orthologs, QS domain LuxR proteins, phylogeny

## Abstract

LuxR solo transcriptional regulators contain both an autoinducer binding domain (ABD; *N*-terminal) and a DNA binding Helix-Turn-Helix domain (HTH; *C*-terminal), but are not associated with a cognate *N*-acyl homoserine lactone (AHL) synthase coding gene in the same genome. Although a few LuxR solos have been characterized, their distributions as well as their role in bacterial signal perception and other processes are poorly understood. In this study we have carried out a systematic survey of distribution of all ABD containing LuxR transcriptional regulators (QS domain LuxRs) available in the InterPro database (IPR005143), and identified those lacking a cognate AHL synthase. These LuxR solos were then analyzed regarding their taxonomical distribution, predicted functions of neighboring genes and the presence of complete AHL-QS systems in the genomes that carry them. Our analyses reveal the presence of one or multiple predicted LuxR solos in many proteobacterial genomes carrying QS domain LuxRs, some of them harboring genes for one or more AHL-QS circuits. The presence of LuxR solos in bacteria occupying diverse environments suggests potential ecological functions for these proteins beyond AHL and interkingdom signaling. Based on gene context and the conservation levels of invariant amino acids of ABD, we have classified LuxR solos into functionally meaningful groups or putative orthologs. Surprisingly, putative LuxR solos were also found in a few non-proteobacterial genomes which are not known to carry AHL-QS systems. Multiple predicted LuxR solos in the same genome appeared to have different levels of conservation of invariant amino acid residues of ABD questioning their binding to AHLs. In summary, this study provides a detailed overview of distribution of LuxR solos and their probable roles in bacteria with genome sequence information.

## Introduction

Bacteria sense and respond to changes in external environments through signal transduction systems that include transcriptional regulators for modulating gene expression. A sub-group of LuxR transcriptional regulators with *N*-terminal autoinducer binding domains (ABD) and *C*-terminal Helix-Turn-Helix (HTH) DNA binding domains are known to be involved in quorum sensing (QS) signaling in many proteobacteria (the presence of both domains is referred to as QS domain here on) (Choi and Greenberg, 1991; Hanzelka and Greenberg, 1995; Luo and Farrand, 1999). Genes coding for these LuxR regulators usually occur together with a gene coding for the synthesis of *N*-acyl homoserine lactone (AHL) signaling molecules, the LuxI homolog. QS typically involves production of AHLs by a LuxI homolog and their sensing by the LuxR regulator in a cell-density dependent manner to regulate target genes (Fuqua et al., 1994; Zhu and Winans, 2001; Fuqua and Greenberg, 2002). Studies in the last 10 years have uncovered a new group of LuxR regulators that occur without the cognate LuxI homolog and they are referred to as LuxR orphans or solos (Fuqua, 2006; Patankar and Gonzalez, 2009b; Subramoni and Venturi, 2009a). LuxR solos have the same domain organization as canonical LuxR proteins of the QS system, and have been found in different bacteria with important roles in processes such as virulence, plant growth promotion, nodulation, motility, plasmid transfer, antibiotic synthesis, and regulation of QS. They are thought to be important for bacterial signal perception in inter-bacterial and host–bacterial interactions (Soares and Ahmer, 2011; Venturi and Fuqua, 2013).

Several studies have led to the view that LuxR solos may bind to AHLs or to other non-AHL molecules and regulate bacterial traits important for fitness in the environment or in association with their hosts. AHL-binding LuxR solos characterized so far include QscR of *Pseudomonas aeruginosa*, SdiA of *Escherichia coli* and *Salmonella typhimurium*, ExpR of *Sinorhizobium meliloti*, BisR of *Rhizobium leguminosarum* bv. *viciae*, VjbR of *Brucella melitensis*, and PpoR of *Pseudomonas putida* (Ahmer et al., 1998; Chugani et al., 2001; Pellock et al., 2002; Wilkinson et al., 2002; Ahmer, 2004; Delrue et al., 2005; Fuqua, 2006; Subramoni and Venturi, 2009b). Non-AHL binding LuxR solos that recognize yet unknown plant-derived molecules have been studied in several plant-associated bacteria and include OryR of *Xanthomonas oryzae* pv. *oryzae*, XccR of *Xanthomonas campestris* pv. *campestris*, PsoR of *P*. *protogenes* and NesR of *S. meliloti* (Ferluga et al., 2007; Zhang et al., 2007; Ferluga and Venturi, 2009; Patankar and Gonzalez, 2009a; Subramoni et al., 2011). A few LuxR solos like CarR of *Serratia marscecens* and CepR2 of *Burkholderia cenocepacia* are also known to regulate target genes in a ligand independent manner (Cox et al., 1998; Malott et al., 2009; Poulter et al., 2011; Ryan et al., 2013). Mostly LuxR solos bind to their ligands and activate expression of their target genes but CarR and CepR2 act as repressors and are known to de-repress target genes in the presence of AHLs.

Similar to QS-associated LuxRs, LuxR solos have been shown to bind to 20-bp palindromic sequences in the promoter regions of genes regulated by them, referred to as “*lux box*” (Devine et al., 1989; Whiteley and Greenberg, 2001; Zhang et al., 2007; Gonzalez et al., 2013). The QS domain LuxR proteins show low sequence similarity (20–25%) but are known to have nine invariant amino acid residues that are critical for ligand and DNA binding properties of these proteins (Whitehead et al., 2001; Zhang et al., 2002). These conserved amino acids are W57, Y61, D70, P71, W85, G113, E178, L182, and G188 with respect to TraR amino acid sequence; the first six amino acids are present in the ABD and the last three amino acids in the HTH domain (Fuqua et al., 1996). The conservation of these key residues is thought to indicate binding of these QS domain LuxRs to AHLs whereas a lack of conservation raises the possibility of binding to other ligands (Patankar and Gonzalez, 2009b).

The availability of an increasing number of bacterial genome sequences has enabled *in silico* analysis for LuxR and LuxI proteins (Sabag-Daigle and Ahmer, 2012). A previous study reported the existence of a much higher number of genes coding for LuxR homologs compared to LuxI homologs in sequenced bacteria suggesting that these genomes might be harboring LuxR solos in addition to canonical LuxRs of QS systems (Case et al., 2008). *In silico* survey of LuxR proteins is complicated by the fact these family of proteins may have different types of domains at the *N*-terminal associated with the *C*-terminal HTH DNA binding domain; one of these domains is the ABD found in QS domain LuxRs. Since only a few LuxR solos have been studied, the distribution, conservation, evolutionary relatedness and functional roles of these interesting group of proteins remains largely unknown.

In this study we have carried out a systematic survey for QS-domain LuxRs in sequenced bacterial genomes included in the Interpro database, and differentiated where possible the putative LuxR solos from LuxR proteins of QS systems. We have also divided several of these LuxR solos into different functionally relevant groups based on their neighboring gene information and determined their relatedness to LuxR solos with known ligands/roles. Our analysis reveals the extent of occurrence of homologs of LuxR solos with known properties and several LuxR solos with probable unknown AHL or non-AHL ligand binding properties. LuxR solos from closely related genomes carrying multiple numbers of these proteins cluster in different sub-groups and have different levels of conservation of amino acids reported to be important for ligand binding. Overall, our analysis has provided a method to classify LuxR solos from sequenced genomes and will enable studies on newly identified members of these type of proteins that are currently being added to the Interpro database.

## Materials and methods

### Identification of LuxR solos and analysis of neighboring loci

The complete collection of LuxR proteins with N-terminal ABD, was obtained from the InterPro database (InterPro entry IPR005143), which contains sequences from member databases, PROSITE, Pfam, Prints, ProDom, SMART, and TIGRFAMs (McDowall and Hunter, 2011; Hunter et al., 2012). As of August 31st, 2014, all protein sequences in IPR005143 with the signature “transcriptional factors LuxR-like, autoinducer-binding domain” were analyzed. The combinations of different domain architectures of the proteins analyzed from this Interpro collection include IPR005143 (ABD)–IPR000792 (Transcription regulator LuxR, C-terminal), IPR005143–IPR016032 (Signal transduction response regulator, C-terminal effector), and IPR005143–IPR011991 (Winged HTH DNA-binding domain). Each protein entry was analyzed to determine the following; the sequencing status of the genome to select only the completed ones, the niche or source of the bacterial isolate (animal, environmental, human or plant), the gene products encoded by the flanking genes and the presence of genes coding for complete QS LuxI/LuxR pairs in the same genome.

The protein entries obtained from Interpro were classified as LuxR solos (1) if no gene coding for a LuxI homolog was found in the genome, (2) if no gene was found in the genomic locus near the gene coding for QS domain LuxR protein, or (3) if no unpaired or extra genes coding for LuxI homolog were present in the genome. The genomes carrying these QS domain LuxRs were assigned to three categories: LuxR solos, LuxR solo + QS (if a LuxI/R pair(s) was encoded in the genome in addition to a LuxR solo protein), or QS (if a LuxI/R pair(s) was encoded in the genome but no LuxR solo protein was found). In those cases where LuxR solo proteins were found, the number of genes coding for LuxR solo proteins in each genome was noted. These data were used to generate contingency tables, by using the dynamic table tool available in Microsoft Office 11 and graphs were plotted from the table data using GraphPad Prism.

### Multiple sequence alignment and analysis for invariant amino acids

LuxR solos were aligned against TraR amino acid sequence, with Clustal W (Thompson et al., 1994, 2002; Larkin et al., 2007). The presence of all nine key residues previously reported to be invariant in several functionally characterized QS LuxR proteins (W57, Y61, D70, P71, W85, G113, E178, L182, G188 with respect to TraR of *Agrobacterium tumefaciens*) (Whitehead et al., 2001; Zhang et al., 2002) was evaluated by inspection of the alignment.

### Sequence alignment and phylogenetic analyses

Evolutionary analyses were conducted using MEGA 6.06 (Tamura et al., 2013). Protein sequences were grouped based on bacterial class, and groups were aligned by MUSCLE (Edgar, 2004). Phylogenetic analyses were performed by using the Maximum Likelihood method based on the JTT matrix-based model (Jones et al., 1992), which generated trees with the highest log likelihood. In each case significance was estimated by using Bootstrap analysis. Initial tree(s) for the heuristic search were obtained automatically by applying Neighbor-Joining and BioNJ algorithms to a matrix of pairwise distances estimated using a JTT model, and then selecting the topology with superior log likelihood value. All positions containing gaps and missing data were eliminated. A group of functionally characterized QS-associated LuxRs and LuxR solo proteins were included in all phylogenetic analyses to determine relatedness (Table 1), and transcriptional regulator GerE from *Bacillus subtilis* was included as outgroup, as this sequence is more distantly related to the LuxR solo sequences than they are to each other (Hall, 2013), and has been included previously in similar phylogenetic analyses (Subramoni et al., 2011; Gonzalez and Venturi, 2013).

**Table 1:** **LuxR solos included as reference in the phylogenetic analyses**.

**LuxR solo protein**	**Species and strain**	**Accession number**
RhlR	*P. aeruginosa* PAO1	AAC44036.1
CviR	*Chromobacterium violaceum* ATCC31532	AAP32919.1
CepR CepR2	*B. cenocepacia* J2315	YP 002234479.1 B4EHM0
PfsR	*P. fuscovaginae* UPB0736	CBI67623.1
SinR	*S. meliloti* SM11	AEH78836.1
LuxR	*Vibrio fischeri* ES114	AAA27542.1
TraR	*A. tumefaciens* AAZ50597.1	AAZ50597.1
LasR	*P. aeruginosa* PAO1	AAG04819.1
PmlR	*B. pseudomallei* K96243	YP 110896.1
RpaR	*Rhodopseudomonas palustris* CGA009	NP 945674.1
BjaR	*Bradyrhizobium diazoefficiens* USDA110	NP 767702.1
PluR	*Photorhabdus luminescens subsp. Laumondii* TT01	AGO97061.1
EsaR	*Pantoea stewartii* subsp. *stewartii* DC283	AAA82097.1
SdiA	*S. enterica* subsp. *enterica* serovar *Typhimurium*	AAC08299.1
CinR	*R. leguminosarum 8401*	AF210630.2
QscR	*P. aeruginosa* PAO1	G3XD77
OryR	*X. oryzae* KACC10331	Q5H3E9
ExpR	*S. meliloti* RU11	W0X916
CarR	*S. marcescen*s ATCC 39006	AAC38168.1
VjbR	*Brucella melitensis* 16M	Q8YAY5.2
Ger E	*Bacillus subtilis* strain 168	CAA11701.1

### Identification of *lux box* and operon prediction

In order to determine the presence of a *lux box* in specific promoters, upstream sequences were retrieved using tools available at RSAT (Thomas-Chollier et al., 2011) and promoter regions identified using BPROM (Solovyev and Salamov, 2011). Twenty base pairs of palindromic sequences in the promoters were then identified using the motif discovery tool of MEME (Bailey et al., 2009). Identified sequences were then aligned with known *lux box* sequences. Operon prediction was carried out using tools available at FGENESB (Tyson et al., 2004).

### Cluster analysis and identification of putative orthologous groups

The entire collection of LuxR solos (almost 5000 proteins) was analyzed by CD-HIT (Huang et al., 2010) to group together all protein sequences that showed sequence identity greater than 90%. This would help to remove very closely related protein sequences from the LuxR solos collection. This reduced sub-set consisting of representative LuxR solo sequence from each group (657 proteins; data not shown) was used for further analysis. In order to identify closely related members among this reduced collection of LuxR solos, CLANS analysis (Frickey and Lupas, 2004) was carried out. CLANS performs BLAST analysis of each sequence against all other sequences individually based on *P*-values of high-scoring segment pairs and enables two-dimensional visualization of pair-wise sequence similarities. For network-based clustering the *P*-value cut off was set to 10^−30^ and the attraction and repulsion exponents were set to two. In another approach to classify closely related LuxR solos into functionally related groups, the neighboring genes flanking the genes coding for representative LuxR solos were analyzed using SynTax (Oberto, 2013). The conservation of invariant amino acids was also checked. LuxR solos with similar flanking genes and similar amino acid conservation were grouped together.

## Results

### Distribution of LuxR solos in sequenced bacterial genomes

The complete collection of QS domain LuxR proteins were sourced from Interpro database (IPR005143) and analyzed to identify LuxR solos or orphans according to the criteria outlined in materials and methods. In total, 6030 QS domain LuxR protein sequences from 3540 sequenced genomes were analyzed and an inventory of 4860 LuxR solos and 1170 LuxR proteins that are part of complete QS systems generated (Supplementary Table 1). Majority of LuxR solos were carried by chromosomal loci but some were encoded by plasmids as found in *Oligotropha carboxidovorans*, *Methylobacterium extorquens*, *Agrobacterium* sp., and *R. leguminosarum* bv. *trifolii* CB782. Genes coding for LuxR solos were sometimes located near a gene coding for transposase as found in *M. australicum*, *R. loti*, *R. leguminosarum* bv. *viciae*, *R. tropici*, *S. meliloti*, *Oceanicola batsensis, Octadecabacter arcticus, Paracoccus aminophilus, Roseivivax isoporae, Gluconacetobacter medellinensis, Erythrobacter sp. SD-21, Novosphingobium sp. PP1Y, B. thailandensis, Acidovorax avenae*, and *Alcanivorax pacificus W11-5*. In some cases it was not possible to delineate a LuxR solo or a canonical QS LuxR from multiple QS domain LuxRs present in a genome; for example (1) when two QS domain LuxR proteins occur in tandem near a gene coding for the LuxI homolog (examples include *Nitratireductor aquibiodomus* RA22, *Agrobacterium tumefaciens* 5A, *Rhodobacter sphaeroides* (strain ATCC 17025/ATH 2.4.3), *Sphingobium baderi* LL03 and *S. lactosutens* DS20, (2) when the gene coding for a LuxI homolog was located in a locus genetically unlinked from the locus coding for a QS domain LuxR homolog or two QS domain LuxR homologs adjacent to each other (as in species belonging to Rhizobiales, Rhodobacteriales, and Burkholderiales), and (3) when truncated LuxR proteins containing only the ABD without the DNA binding domain were present in a genome; genes coding for these proteins were often located near gene(s) coding for a QS domain LuxR protein (Supplementary Table 2). Adjacently located genes coding for two LuxR solos may also occur in genomes without an unpaired LuxI homolog as found in several bacteria belonging to Burkholderiales and Rhizobiales. These are described in more detail later in the Results section.

**Figure 1:**
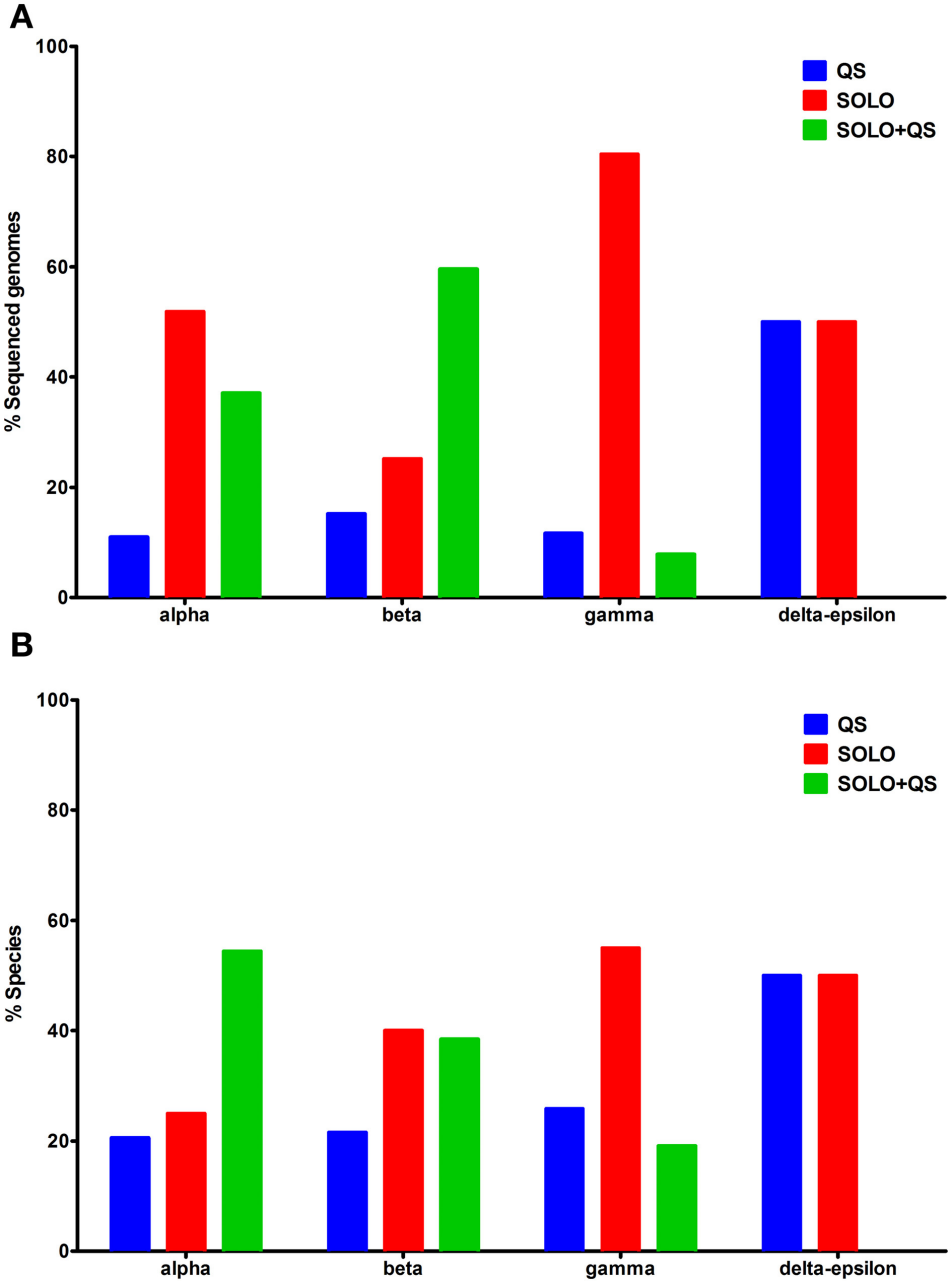
**Distribution of QS domain LuxRs in different bacterial classes. (A)** Percentage of sequenced genomes carrying QS domain LuxR proteins from each class, having QS LuxRs alone, LuxR solos alone, or both **(B)** Percentage of species carrying QS domain LuxR proteins from each class, having QS LuxRs alone, LuxR solos alone, or both. QS domain LuxR protein sequences were sourced from Interpro database, IPR005143. On the x-axis, alpha-Alphaproteobacteria, beta-Betaproteobacteria, gamma-Gammaproteobacteria, and delta-epsilon-Delta-Epsilonproteobacteria.

The taxonomic distribution of LuxR solo proteins in sequenced bacterial genomes was found to be biased due to the availability of a larger number of sequences for some bacterial species with clinical or agricultural importance (Figure 1A). For example, a larger number of sequenced genomes are available for Alphaproteobacteria and Gammaproteobacteria species that carry only LuxR solos. However, examination of LuxR solo occurrence at species level was more representative of actual numbers and distribution (Figure 1B). QS domain LuxR proteins were found to be mainly restricted to proteobacteria as reported previously (Case et al., 2008); surprisingly a few non-proteobacterial sequenced genomes were also found to carry these proteins (discussed below). Among proteobacteria carrying QS domain LuxRs, 10–15% of sequenced genomes representing 20–25% of bacterial species carried only complete QS systems without any additional QS domain LuxR proteins (Figures 1A,B; Supplementary Table 1). On the other hand, majority of bacteria (approximately 75% at species level) harbor one or more LuxR solo or orphan proteins, either with or without complete QS system(s). In Alphaproteobacteria more than 50% of sequenced genomes representing 25% of species carry LuxR solos alone whereas 37% of sequenced genomes constituting 54% species carry both LuxR solos and complete QS systems. In Betaproteobacteria, the numbers of species that contain LuxR solos alone or LuxR solos in addition to complete QS systems are similar although more genomes have been sequenced for the latter. In contrast to this, 80% of Gammaproteobacterial genomes representing 54% species carry only LuxR solos whereas 20% carry both LuxR solos and QS system(s) (Figures 1A,B). Very few genomes belonging to Delta-Epsilonproteobacteria carry QS domain LuxR proteins; of those, 50% carry complete QS systems and the rest carry only LuxR solos. Bacteria belonging to *Brucella* sp., *Ochrobactrum* sp., *Acidovorax* sp., *Citrobacter* sp., *Cronobacter* sp., *Escherichia* sp., *Salmonella* sp., *Enterobacter* sp., *Shigella* sp., *Yersinia* sp., *Xanthomonas* sp. as well as several *Pseudomonas* sp. were among those that were found to carry LuxR solos alone without any QS system. Several species that belonged to *Rhizobium* sp., *Mesorhizobium* sp., *Agrobacterium* sp., *Burkholderia* sp., *Aeromonas* sp., *Serratia* sp., and several *Pseudomonas* sp. carry both complete QS systems as well as genes for LuxR solos. A complete list is provided in Supplementary Table 1. The most varied distribution in terms of both presence and numbers of genes coding for LuxR solos and complete QS systems was found in bacteria belonging to *Bradyrhizobium* sp., *Methylobacterium* sp., *Serratia* sp., *Yersinia* sp., and *Pseudomonas* sp.

### Bacteria carrying LuxR solos occur in diverse ecological niche(s)

**Figure 2:**
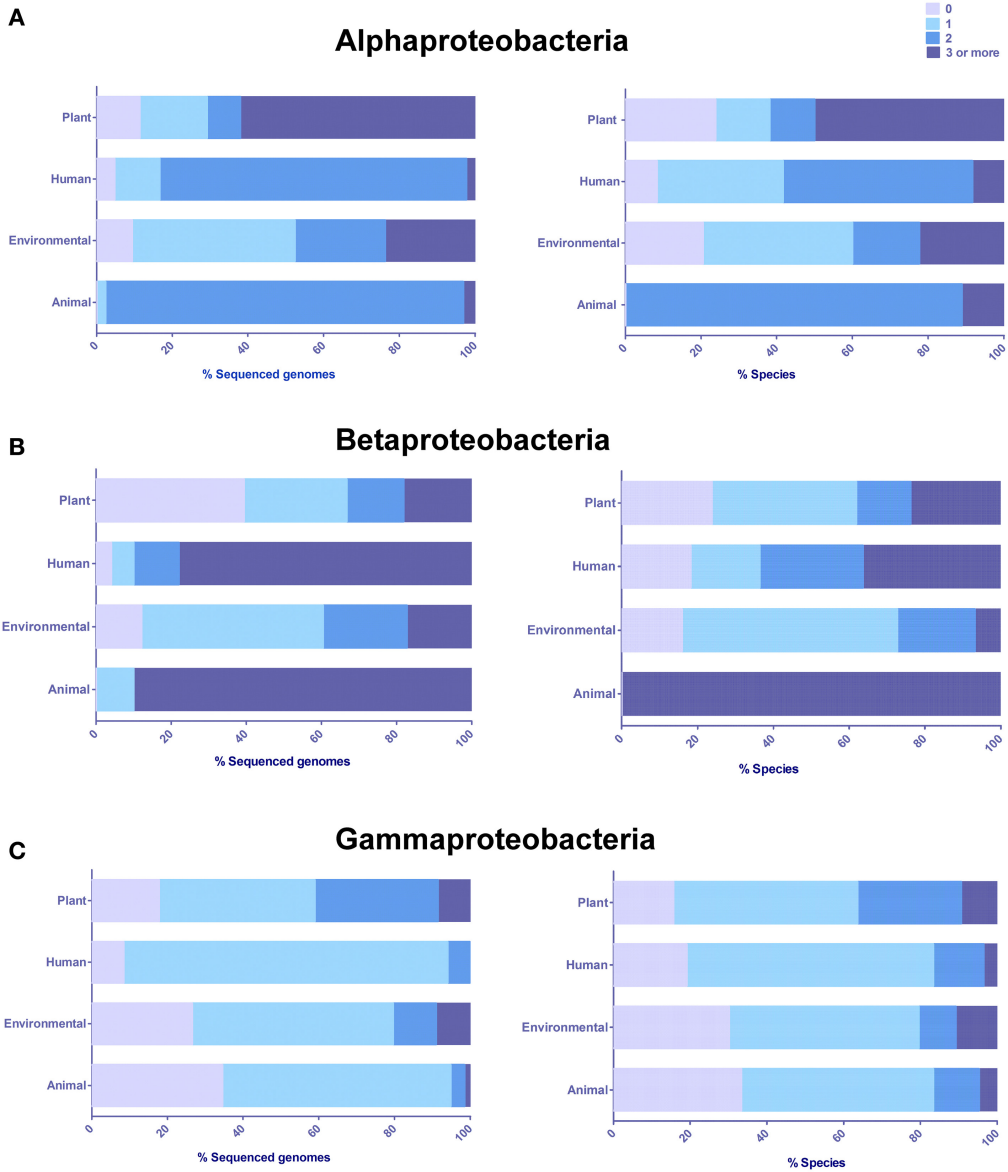
**Distribution and abundance of LuxR solos in different classes based on the origin or predominant niche specificity of bacterial species harboring these proteins**. **(A)** Alphaproteobacteria **(B)** Betaproteobacteria, **(C)** Gammaproteobacteria. Plant, Plant-associated bacteria; Human, Human-associated bacteria; Environmental, Environmental isolates; and Animal, Animal-associated bacteria. Some bacterial species were placed in more than one category. The presence of LuxR solos was inferred by analyzing QS domain LuxR proteins of Interpro database, IPR005143, and correlated to niche specificity by generating contingency tables in Microsoft Excel.

In order to determine if there was any taxonomic- or niche-specific trend for occurrence of LuxR solos, an analysis of abundance of these proteins with respect to various taxa and ecological niche of bacterial species that harbor them was carried out (Figure 2). The number of genes coding LuxR solos in a bacterial genome ranged from one to as high as seven (described below). In Alphaproteobacteria, plant-associated species mostly carried multiple LuxR solos whereas human and animal-associated species typically carried two LuxR solos. A large proportion of environmental isolates belonging to Alphaproteobacteria carried one LuxR solo but a substantial number of species also carried two or more LuxR solos (Figure 2A). Among bacteria belonging to Betaproteobacteria, majority of plant-associated and environmental isolates carried one LuxR solo; animal and human associated Betaproteobacteria mostly carried multiple LuxR solos (Figure 2B). Exceptions include plant pathogens *B. gladioli* and *B. glumae* harboring multiple solos. Majority of bacteria belonging to Gammaproteobacteria carried one LuxR solo irrespective of whether they were environmental or associated with plant, human or animal hosts (Figure 2C). In each of the three taxonomic groups a large number of plant-associated and environmental isolates carried multiple LuxR solos; overall, these observations suggest a role for multiple LuxR solos in bacterial species occupying environmental and/or plant-associated niche.

### LuxR solos are also found in a few sequenced non-proteobacteria

Our analysis of the Interpro database for QS domain LuxR proteins revealed the presence of 11 proteins with this domain architecture in non-proteobacterial genomes. Since a gene coding for a LuxI homolog could not be identified by examination of these non-proteobacterial genome sequences, these proteins were considered as LuxR solos. These were distributed in Actinobacteria (six LuxR solos), Chlamydiae/Verrucomicrobia (two LuxR solos), Nitrospirae (two LuxR solos), and Chrysiogenetes (one LuxR solo), respectively (Supplementary Table 1).

**Figure 3:**
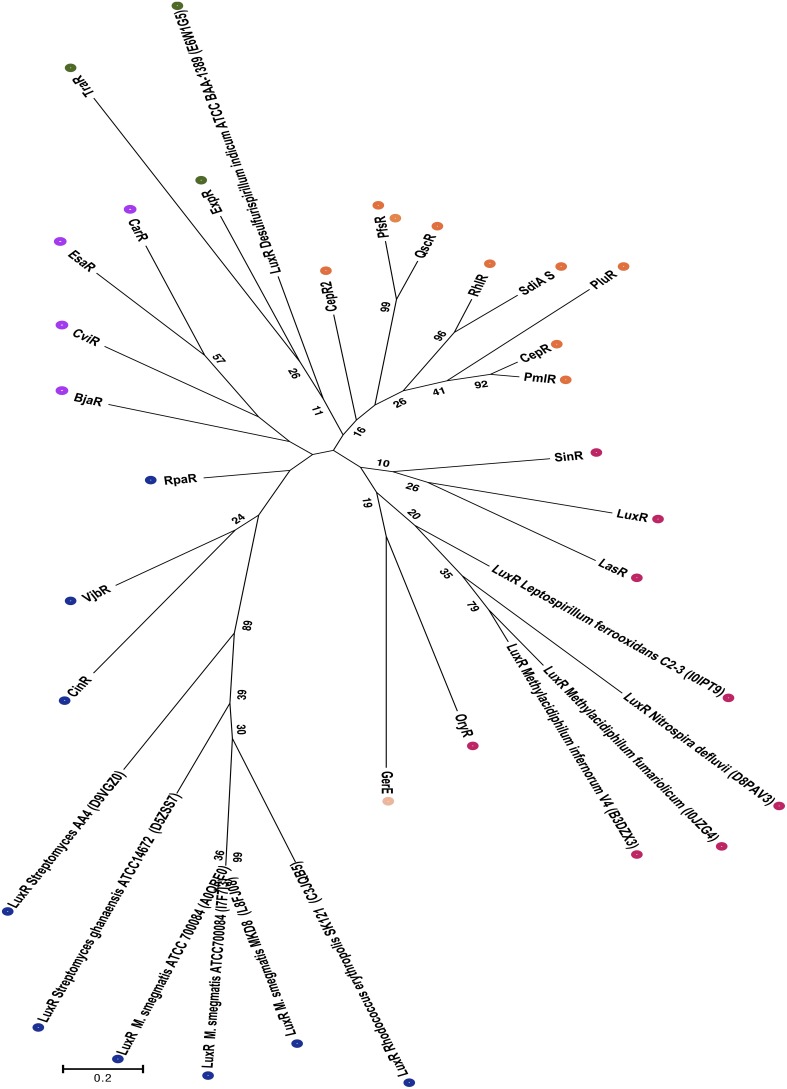
**Phylogenetic analyses of non-proteobacterial LuxR solos**. This evolutionary history was inferred by using the Maximum Likelihood and the unrooted tree with the highest log likelihood is shown. The tree is drawn to scale, with branch lengths measured in the number of substitutions per site, and colored dots indicate different groups as discussed in the results section. The analysis involved 33 amino acid sequences, which included the 11 Non-proteobacterial LuxR solos indicated by their Uniprot identification in the figure. All positions containing gaps and missing data were eliminated. There were a total of 71 positions in the final dataset. Numbers in brackets indicate UNIPROT accession numbers for all proteins analyzed.

In order to determine the evolutionary relatedness of non-proteobacterial LuxR solos to Gram-negative canonical QS LuxRs and LuxR solos an unrooted phylogenetic tree was generated as described in methods (Figure 3). Four major branches could be delineated in the phylogenetic tree; (1) The Actinobacterial (*Mycobacterium* sp., *Streptomyces* sp., and *Rhodococcus* sp.) LuxR solos form a robust clade that branches out from rest of the tree. Three other QS domain LuxRs, namely, RpaR, CinR, and VjbR form another group of this branch. (2) The LuxR solos of *Methylacidiphilum fumariolicum*, *M. infernorum*, *Nitrospira defluvii*, and *Leptospirillum ferrooxidans* form another separate group clustering together with LasR, LuxR, SinR, and OryR suggesting that they may be evolutionarily closer to these QS domain LuxRs when compared to Actinobacterial LuxR solos. (3) The third branch of the tree is made up of functionally characterized proteobacterial QS domain proteins CarR, EsaR, CviR, and BjaR, indicating high evolutionary relatedness of these proteins. (4) In the fourth major clade of the tree, a LuxR solo of *Desulfurispirillum indicum* was found to cluster with TraR and ExpR; the branching of this LuxR solo also revealed a shared common ancestor with other QS LuxR and LuxR solos like CepR2, PfsR, QscR, RhlR, SdiA, PluR, CepR, and PmlR. In summary, with respect to well-studied proteobacterial QS LuxRs and LuxR solos, the Actinobacterial LuxR solos are evolutionarily distant whereas other non-proteobacterial LuxR solos show a relatively higher degree of evolutionary relatedness.

It was of interest to analyze the sequence features of ligand binding domains of non-proteobacterial LuxR solos as there are no reported studies regarding these proteins. Our analysis revealed lack of conservation in at least two/nine amino acids in all these proteins; importantly the amino acid corresponding to W85 of TraR was changed (Table 2). The LuxR solos of *M. fumariolicum* and *M. infernorum* carry W57L and W85R substitutions at the corresponding positions in their proteins. The LuxR solos of *Mycobacterium* sp., *Streptomyces* sp., and *Rhodococcus* sp. carry W57_ and W85D or R substitutions. *D. indicum* and *N. defluvii* solos also carry W85R or W85_ and W57F substitutions. The lack of conservation of these invariant amino acids raises the possibility that these LuxR solos may bind to ligands different from AHLs.

**Table 2:** **Conservation analyses for LuxR-solo proteins found in non-proteobacterial genomes**.

**LuxR proteins found in non-proteobacterial genomes**		**Key aminoacids in autoinducer binding domain**						**Key aminoacids in HTH domain**		
**Accession number**	**Species**	**W**	**Y**	**D**	**P**	**W**	**G**	**E**	**L**	**G**
		**57**	**61**	**70**	**71**	**85**	**113**	**178**	**182**	**188**
I0JZG4	*Methylacidiphilum fumariolicum*	L	Y	D	P	R	G	E	I	G
B3DZX3	*Methylacidiphilum infernorum* V4	L	Y	D	P	R	G	E	L	G
I7F7I3	*Mycobacterium smegmatis* ATCC700084	gap	Y	K	E	D	G	E	L	G
A0QRE0	*Mycobacterium smegmatis* ATCC700084	gap	Y	K	P	D	G	E	L	G
D9VGZ0	*Streptomyces sp. AA4*	gap	Y	C	P	R	G	E	L	G
D5ZSS7	*Streptomyces ghanaensis* ATCC 14672	gap	Y	D	P	W	G	E	L	G
C3JQB5	*Rhodococcus erythropolis*	gap	Y	D	P	R	G	E	L	G
L8FJ08	*Mycobacterium smegmatis* MKD8	gap	Y	D	P	D	G	E	L	G
E6W1G5	*Desulfurispirillum indicum* strain ATCC BAA-1389	W	Y	D	P	R	G	E	L	G
D8PAV3	*Nitrospira defluvii*	F	Y	D	P	gap	C	E	L	G
I0IPT9	*Leptospirillum ferrooxidans* strain C2-3	W	Y	D	P	gap	G	E	L	G

*Each entry was aligned against TraR from Agrobacterium tumefaciens*.

### Cluster analysis of LuxR solos

**Figure 4:**
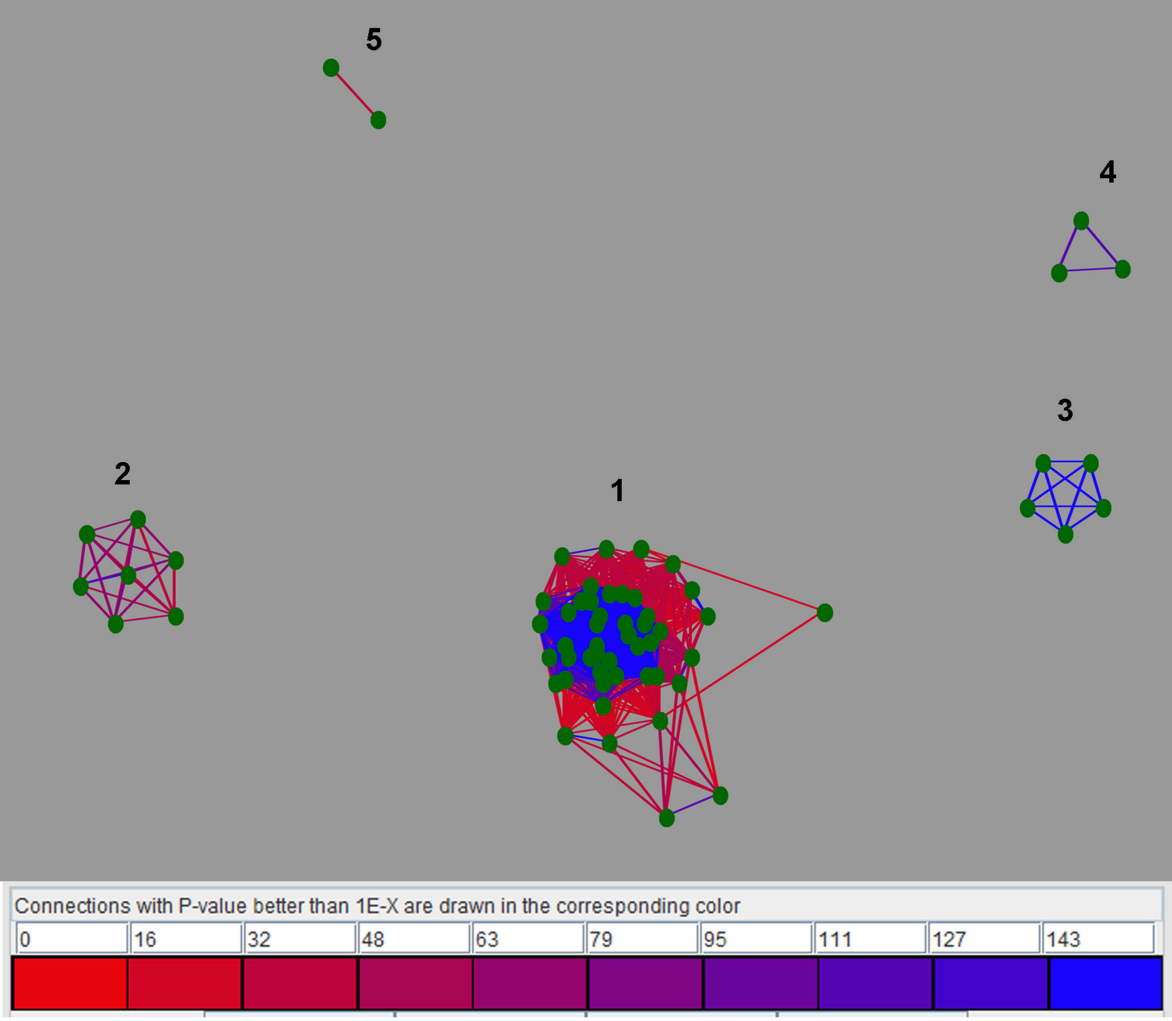
**Two-dimensional CLANS clustering of LuxR solos**. CLANS analysis was carried out on the representative 657 LuxR solo sequences as detailed in materials and methods. LuxR solos that clustered are represented as green dots and unclustered proteins are not shown. BLAST sequence similarities are indicated by lines shaded from red (*P*-values < 10^−35^), to blue (*P*-values < 10^−200^. Different clusters are indicated by numbers.

In order to group related LuxR solos, cluster analysis was carried out using CLANS as detailed in materials and methods. Results using the sub-set of 657 LuxR solo proteins revealed that majority of the LuxR solos remained ungrouped probably due to low sequence similarity. Only a small number (65 out of 657 LuxR solos) clustered together mainly into five groups; these groups are likely a reflection of their taxonomic relationships. Among these, only LuxR solos from closely related species showed conservation of flanking genes (Figure 4). These clusters are as follows; cluster 1 consisting of 47 LuxR solos of mixed taxonomy with varying levels of relatedness (as indicated by the differently colored lines), cluster 2 consisting of seven LuxR solos mainly Betaproteobacteria, cluster 3 consisting of five LuxR solos, cluster 4 with three LuxR solos and cluster 5 with two sequences (Supplementary Table 3). Cluster 1 was formed by LuxR solos of Alphaproteobacteria (*Agrobacterium* sp., *Sphingomonas* sp., *Roseivivax* sp., *Sagittula* sp., *Nitratireductor* sp., *Methylobacterium* sp., *Aurantimonas* sp., *Afipia* sp., *Paracoccus* sp., *Sinorhizobium* sp., *Rhodospirillum* sp., *Pelagibaca* sp.), and Betaproteobacteria (*Burkholderia* sp., *Variovorax* sp., *Chromobacterium* sp.), showing high degree of similarity within each group but lesser similarity between them. A few LuxR solos of Gammaproteobacteria represented by *Dickeya* sp., *Klebsiella* sp., *Alcanivorax* sp., *Pseudomonas* sp., as well a LuxR solo from *Streptomyces* were also in cluster 1. *Burkholderia* sp. LuxR solos were distributed in all clusters. A step-wise increase in *P*-value cut off from 10^−30^ to 10^−1^ resulted in merging of these small clusters into a single cluster suggesting that these 65 LuxR solos are closely related. Decreasing *P*-value to 10^−35^ resulted in separation of clusters and at *P*-value of 10^−200^ all proteins separated and remained independent without any cluster formation. These results confirm the fact that LuxR solos from different taxonomic groups have low levels of sequence relatedness making it very difficult to compare across bacterial taxa.

### Functional grouping of LuxR solos

In order to group LuxR solos in a biologically relevant manner, an alternate approach was used whereby the subset of representative LuxR solos were analyzed for the genomic context of genes encoding them and the conservation of invariant amino acids of ABD. By this approach LuxR solos could be divided into different groups consisting of known and unknown LuxR solos; members of each group are likely to be orthologs (Tables 3, 4). Out of the 657 representative LuxR solos, 272 could be placed into these categories; 385 remained ungrouped using these criteria (Supplementary Tables 4, 5).

**Table 3:** **Groups of known LuxR solos**.

Group Known LuxR solo/LuxRI^*^	**Genomic context of LuxR solo**	**Bacterial species**	**Homology, Invariant amino acids changed, Reference/(s)**
SdiA (*S. typhimurium and E. coli*)	*yecC, ABC transporter –* ***sdiA***– *yecF, Uncharacterized protein (C) – sirA, Invasion response regulator – uvrC, UvrABC system protein C*	*Enterobacter sp*., *Klebsiella sp*., *Cronobacter sp*., *Escherichia sp*., *Yokenella sp*., *Citrobacter sp*., *Salmonella sp*., *Shimwellia sp*.	66–82%
ExpRI (*Erwinia*), PhzRI (*Pantoea*)			None
			Ahmer et al., 1998; Kanamaru et al., 2000; Michael et al., 2001; Sabag-Daigle and Ahmer, 2012
QscR (*P. aeruginosa*)	*Desaturase (C) –* ***qscR***– *phzA, phenazine biosynthesis protein A*	*P. aeruginosa*	97–100%
			None
			Chugani et al., 2001; Oinuma and Greenberg, 2011
XccR (*X. campestris*), OryR (*X. oryzae*), PsoR (*P. protegens*), NesR (*S. meliloti*)	*pip, proline imino peptidase/tra, peptide transporter (C) –* ***xccR/oryR/psoR*** – *pip, proline imino peptidase/hp, hypothetical protein*	*Pseudomonas sp*., *Rhizobium sp*., *Xanthomonas sp*., *Klebsiella sp*., *Rhodobacter sp*., *Agrobacterium sp*., *Mesorhizobium sp*., *Citreicella sp*., *Rahnella sp*., *Yersinia sp*., *Brennaria sp*., *Serratia sp*.	43–90%
			W57M, Y61W
			Zhang et al., 2007; Ferluga et al., 2007; Patankar and Gonzalez, 2009a; Subramoni et al., 2011
ExpR (*S. melilotii*), AviR (*A*. *vitis*)	*chvA*, *glucan exporter ATP-binding protein (C) –* ***expR*** – *pyc, pyruvate carboxylase*	*Agrobacterium sp*., *Ensifer sp*., *Rhizobium sp*., *Shinella sp., Sinorhizobium sp., Hoeflea sp*.	40–93%
			None
			Pellock et al., 2002; Zheng et al., 2003; Gao et al., 2014
PpoR (*P. putida*)	*fprB, flavodoxin reductase (C) –* ***ppoR*** – *rlmG, 16S RNA, methylase RsmC*	*P. putida, P. fluorescens, P. synxantha, P. moraviensis, P. brassicacearum, P. plecoglossicida, P. entomophila, P. mosselii*	40–79%
			None
			Subramoni and Venturi, 2009b; Fernandez-Pinar et al., 2011
CepR2 (*B. cenocepacia*)	*araC, AraC family transcriptional regulator –* ***cepR2*** – *geneX, any gene product (C)*	*Burkholderia sp*., *Variovorax sp*., *Caulobacter sp*.	30–94%
			W85A, G113N, E178Q
			(only some proteins) Malott et al., 2009; Ryan et al., 2013
VjbR (*B. melitensis*)	*hp, hypothetical protein –* ***vjbR*** – *tetR, TetR family transcriptional regulator (C)*	*Brucella sp., Ochrobactrum sp., Phyllobacterium sp*.	47–88%
			W85I/V, G113F
			Delrue et al., 2005; Uzureau et al., 2010
BlxR (*B. melitensis*)	*ms, Methionine synthase –* ***blxR*** – *at, Amino transferase*	*Brucella sp., Ochrobactrum sp*.	78–94%
			None
			Rambow-Larsen et al., 2008
AvhR (*A*. *vitis*)	*Calcium binding protein (C) –* ***avhR*** – *nrt, nucleotide binding ABC transporter*	*Agrobacterium sp*.	56–67%
			W57F, D70S, W85R
			Hao et al., 2005
BisR (*R. leguminosarum*)	*trbI, conjugal transfer protein TrbI –* ***bisR*** – *traR, Conjugal transfer regulator TraR*	*Rhizobium sp*.	59–87%
			None
			Wilkinson et al., 2002; Danino et al., 2003
CarR (*S. marcescens*)	*geneX, any gene product –* ***carR*** *– carA, carbapenem biosynthesis protein*	*Serratia sp*.	59–62%
			W57C
			Cox et al., 1998; Poulter et al., 2011

*(C)-Gene in the complementary strand*.

* *indicates presence of LuxRI in a similar genomic context. Bold values indicate LuxR solos*.

**Table 4:** **Groups of unknown LuxR solos**.

**Group**	**Genomic context of LuxR solo (5′–3′)**	**Bacterial species**	**Homology (aa), Invariant amino acids changed**
1	*aroQ, 3-dehydroquinate dehydratase II/geneX, any gene product –* ***luxR solo*** – *tsf, Translation elongation factor Ts (C) – rpoB, RNA polymerase (C)*	*Roseobacter sp*., *Phaeobacter sp*., *Salipiger sp*., *Jannaschia sp*., *Roseibacterium sp*., *Dinoroseobacter sp*., *Pelagibaca sp*., *Sagittula sp*., *Citreicella sp*., *Roseovarius sp*., *Ruegeria sp*., *Oceanibulbus sp*., *Leisingera sp*., *Silicibacter sp*., *Thalassobacter sp*., *Loktanella sp*., *Maritimibacter sp*., *Rhodobacter sp*., *Defluviimonas sp*., *Octadecabacter sp*., *Roseivivax sp*., *Paracoccus sp*., *Celeribacter sp*., *Rubellimicrobium sp., Actibacterium sp*.	58–80%
			W57Y, Y61F, D70I/G/A, P71V, E178Q
2	*CR, crotonyl-CoA reductase –* ***luxR solo*** – *ATPase, helicase, ATP-dependent*	*Roseovarius sp., Oceanibulbus sp., Roseobacter sp., Ruegeria sp. raiR/raiI homologs of P. gallaeciensis, P. inhibens, L. methylohalidivorans, R. pomeroyi, R. denitrificans, R. litoralis, O. antarcticus and D. shibae are flanked by CR*	44–46%
			None
3	*acdH, acyl coA dehydrogenase (C) – merR, MerR family transcriptional regulator (C) –* ***luxR solo*** – *hp, hypothetical protein – merR, MerR family transcriptional regulator (C)*	*Roseobacter sp., Sulfitobacter sp., Oceanibulbus sp*.	46–58%
			W57F, D70S (some proteins)
4	*fliG, flagellar motor switch protein (C) – [hp, hypothetical protein (C) – abd, autoinducer binding domain protein (C)] –* ***luxR solo*** – *asl, Adenylosuccinate lyase (C)*	*Rubellimicrobium sp., Loktanella sp. luxR/luxI homologs of O. antarcticus is flanked by fliG*	53–55%
			None
5	*tk, thymidine kinase (C) –* ***luxR solo*** – *rimO, Ribosomal protein S12p Asp methylthiotransferase (C)*	*Salpiger sp., Roseivivax sp*.	60–62%
			Y61E/H, D70W, P71S, W85L/V/I, E178Q
6	*fabG2, 3-oxoacyl-[acyl-carrier-protein] reductase (C) – iclR, IclR family transcriptional regulator –* ***luxR solo*** – *[***luxR solo***] – mrp, ATP binding Mrp protein*	*Rhizobium sp*., *Agrobacterium sp*., *Ensifer sp., Sinorhizobium sp., Shinella sp*.	48–94%
			40% (adjacent LuxR solos)
			W57F/L, D70S, P71T W85Y/V/N/H, G113S/F/C/A
7	*fliF, flagellar M-ring protein – luxR, LuxR superfamily transcriptinal regulator –* ***luxR solo*** – *hp, hypothetical protein (C) – flhB, flagellar biosynthetic protein (C)*	*Sinorhizobiumm sp., Mesorhizobium sp., Rhizobium sp., Aquamicrobium sp*.	60–89%
			W57Y, Y61C/A, D70E, P71E/D
8	*nramp, Natural resistance-associated macrophage protein (C) –* ***luxR solo*** – ***luxR solo*** *(C) – [t3ss, Type 3 secretion genes (C)] – as, asparagine synthase (C)*	*B. cenocepacia, B. multivorans, B. ambifaria, B. thailandensis, B. vietnamiensis, B. dolosa*	66–90%
			33% (adjacent LuxR solos)
			W57Y/F, Y61F, E178Q
9	*geneX, any gene product –* ***luxR solo*** *(C) – hchA, HchA chaperone protein (C)*	*Pseudomonas sp*., *Acinetobacter sp*., *Vibrio sp*., *Agrobacterium sp*., *Beijerinckia sp*., *Comamonas sp*., *Stenotrophomonas sp., Serratia sp*.	46–98%
			Y61F/T/S/I, D70A/R/Q/N, P71T/R/D/K
10	*HAD, HAD-super family hydrolase –* ***luxR solo*** – *chp, conserved hypothetical protein (C)*	*P. syringae, P. avellanae, P. viridiflava, P. cichorii*	54–99%
			None
11	*hadH, 3-hydroxyacyl-CoA dehydrogenase (C) –* ***luxR solo*** – *uspG, universal stress protein*	*S. marcescens, S. liquifaciens, S. plymuthica*	77–97%
			W57Y
12	*geneX, any gene product –* ***luxR solo*** *(C) – as II, anthranilate synthase II (C) – as I, anthranilate synthase I (C)*	*P. syringae, P. brassicacearum, P. fluorescens*	45–86%
			None

*(C)-Gene in the complementary strand*.

Some LuxR solos fell into groups, which had one or more members already well-studied (Table 3; Supplementary Table 4). Known groups contained putative orthologs of SdiA, QscR, XccR/OryR, ExpR/AviR, PpoR, CepR2, AvhR, CarR, BlxR, and VjbR. Of these, orthologs of XccR/OryR had the broadest taxonomic distribution as they are present in bacteria belonging to Alphaproteobacteria and Gammaproteobacteria. Orthologs of SdiA (Enterobacteriales), QscR (*P. aeruginosa*) ExpR (Rhizobiales), CepR (Burkholderiales), AvhR (Rhizobiales), CarR (*Serratia* sp., *Yersinia* sp.), VjbR and BlxR (*Brucella* sp. and *Ochrobactrum* sp.), and PpoR (*Pseudomonas* spp) were more restricted in their taxonomical distribution in our analysis based on flanking gene conservation. As expected SdiA, PpoR, BlxR, ExpR group members showed no changes in the invariant amino acids of the ABD whereas XccR/OryR, VjbR, and some members of CepR2 group showed replacement of amino acids at positions W57, Y61, W85, and G113 with respect to TraR amino acid sequence. Since there are several studies describing the representative LuxR solos of groups mentioned above, and their functions, they will not be discussed further here.

Among the representative members of uncharacterized LuxR solos seven different groups belonging to Alphaproteobacteria, one group belonging to Betaproteobacteria and three different groups belonging to Gammaproteobacteria, respectively were identified (Table 4; Supplementary Table 5). Of these, only two ortholog groups each belonging to Alphaproteobacteria and Gammaproteobacteria had LuxR solos without changes in the invariant amino acids of the ABD suggesting that these proteins are likely bind to AHLs. In support of this observation it was found that related species carry orthologs of these LuxR solos as part of a complete QS system as seen for groups 2 and 4 (Table 4). One ortholog group contained genes coding for LuxR solos always flanked by a gene coding for HchA chaperone protein and occurred in both Alphaproteobacterial and Gammaproteobacterial species. Surprisingly, most sequenced strains of *P. aeruginosa* were found to contain an ortholog of this LuxR solo, in addition to the well-studied QscR, which appears to be restricted to *P. aeruginosa* strains (Supplementary Table 1). Two ortholog groups (groups 6 and 8 representing Rhizobiales and Burkholderiales) had two genes coding for LuxR solos located adjacently; these LuxR solos showed 30–40% homology to each other and varying levels of substitutions in the invariant amino acids of ABD. For the ortholog group of Rhizobiales, a gene coding for a single LuxR solo could be identified in the same genomic context for some species suggesting that genes coding for LuxR solos in tandem probably arose by gene duplication.

In order to determine probable roles of LuxR solos and flanking genes, transcriptional organization and putative functions encoded by these genes was analyzed in representative genomes of each group (Table 4). The gene coding for 3-dehydroquinate dehydratase, *aroQ*, was always found in the same transcriptional unit as the gene coding for group 1 LuxR solo suggesting a role for this LuxR solo in aromatic amino acid biosynthesis. Similarly, the gene coding for an ATP-dependent exoDnase/helicase, an enzyme involved in DNA metabolism was found in an operon with the gene coding for group 2 LuxR solo upstream of it. A gene coding for a small hypothetical protein was found downstream of group 3 LuxR solo gene in the same transcriptional unit; function of this hypothetical protein is not known although the genes nearby code for lipid metabolism-related functions. Genes coding for group 4, group 5, and group 6 LuxR solos (both single and two adjacent genes) were in separate transcriptional units. Group 4 LuxR solos occur near genes coding for flagellar motor protein, FliG, which is required for motility. Group 5 LuxR solo gene is flanked on either side by genes coding for thymidine kinase and a ribosomal methyl transferase, both enzymes involved in nucleic acid metabolism. Located upstream of the gene coding for LuxR solo of group 6 is *iclR*, encoding an IclR family of transcriptional regulator; these regulators are known to regulate degradation of QS signals, plant-bacterial interaction and secondary metabolite production (Molina-Henares et al., 2006). Genes coding for Group 7 LuxR solos occur as part of a transcriptional unit with a gene coding for LuxR transcriptional regulator (lacking ABD) whose function is unknown. The two genes coding for group 8 LuxR solos are located adjacent to each other as separate transcriptional units and have either convergent or divergent orientation depending on bacterial species. In some *Burkholderia* sp. they were found adjacent to a gene coding for asparagine synthase whereas in others the type 3 secretion genes were inserted in this locus. Genes coding for group 9 LuxR solos and HchA chaperone protein occur in a single transcriptional unit. Gene coding for group 10 LuxR solo is a single transcriptional unit and present downstream of an operon involved in methionine metabolism. The gene coding for group 11 LuxR solo was located adjacent to an operon of five genes coding for fatty acid metabolism functions. Finally the gene coding for group 12 LuxR solo is part of an operon that codes for enzymes of phenyl alanine/tyrosine metabolism. These observations reveal that genes coding for some of these LuxR solos may be transcriptionally linked with neighboring genes pointing to a role in different metabolic pathways.

### Phylogenetic analysis of multiple LuxR solos in a bacterial genome

**Figure 5:**
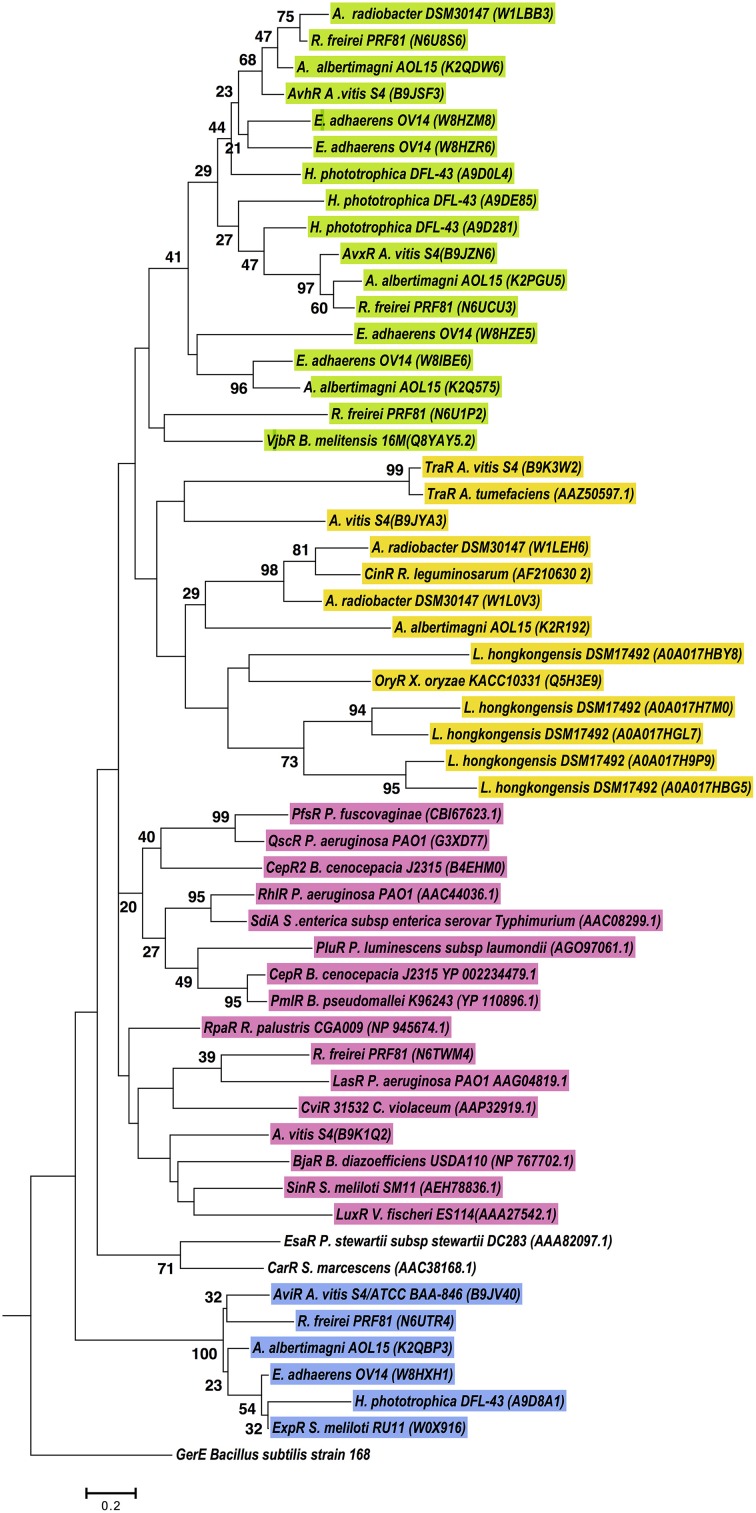
**Phylogenetic analyses of multiple LuxR solos carried by selected Alphaproteobacterial species**. The tree was inferred by using the Maximum Likelihood method. Tree is drawn to scale, with branch lengths measured in the number of substitutions per site. The analysis involved 55 amino acid sequences, from representative species of this class carrying multiple LuxR solos. All positions containing gaps and missing data were eliminated. There were a total of 71 positions in the final dataset. Colors indicate major clusters.

According to our results, several proteobacteria species have more than three genes coding for LuxR solos. In order to determine the relatedness between LuxR solos carried by the same genome, phylogenetic analyses was carried out as detailed in materials and methods. Several species were included for the phylogenetic analyses of these proteins in Alphaproteobacteria (Figure 5). Several clusters were formed, but in most of the cases, LuxR solos from the same strain clustered together in the same branch of the tree indicating high relatedness among them, most likely associated with duplication events, as it evident for LuxR solos from *L. hongkongensis* DSM17492, *E. adherens* OV14 (green clade) and *H. phototrophica* DHL-43 (yellow clade). Interestingly, one independent clade supported by high bootstrap values stems out from this tree and this clade includes ExpR and LuxR solos from *Agrobacterium, H. phototrophica*, *E. adherens* and *R. freirei*. In contrast most of the characterized LuxR proteins locate in a distinct branch suggesting lower levels of relatedness among these proteins and Alphaproteobacterial LuxR solos.

**Figure 6:**
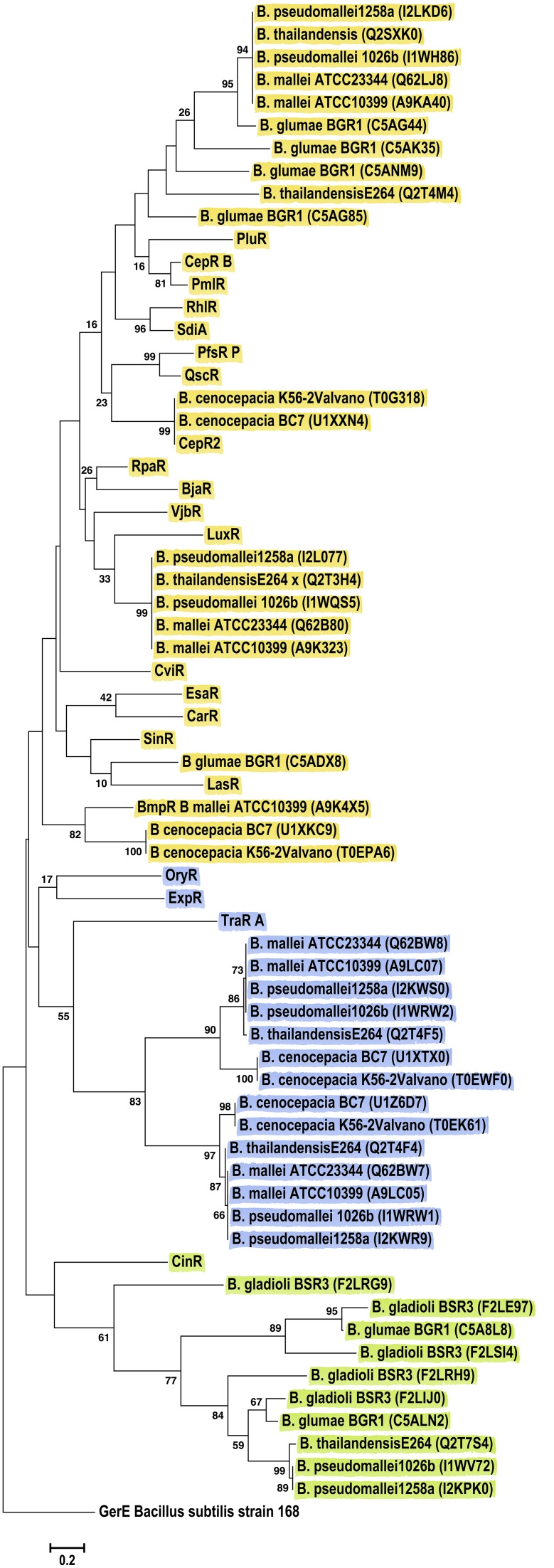
**Phylogenetic analyses of multiple LuxR solos carried by the Betaproteobacteria**. The tree was inferred by using the Maximum Likelihood method. Tree is drawn to scale, with branch lengths measured in the number of substitutions per site. The analysis involved 67 amino acid sequences, representative from the species of the class. This included 46 Betaproteobacterial LuxR solos represented by their Uniprot identification in the figure, in brackets. All positions containing gaps and missing data were eliminated. There were a total of 70 positions in the final dataset. Colors indicate major clusters.

LuxR solos from chosen *Burkholderia* species formed three major clades (Figure 6). Sequences from the *B. mallei–B. pseudomallei* species group were present in all clusters suggesting that these species harbor LuxR solos of different origins. One cluster was mostly composed of proteins from plant-associated strains belonging to the *B. glumae* and *B. gladioli* species, which formed a clade together with CinR (green group Figure 6). Another clade was formed by LuxR solos of *B. mallei*, *B. pseudomallei*, *B. thailandensis* and *B. cenocepacia* that shared an ancestor with TraR, OryR and ExpR (blue group Figure 6). The last clade was composed of LuxR solos that clustered with characterized QS domain LuxRs, most of them known to bind AHLs, forming different sub-groups based on relatedness.

**Figure 7:**
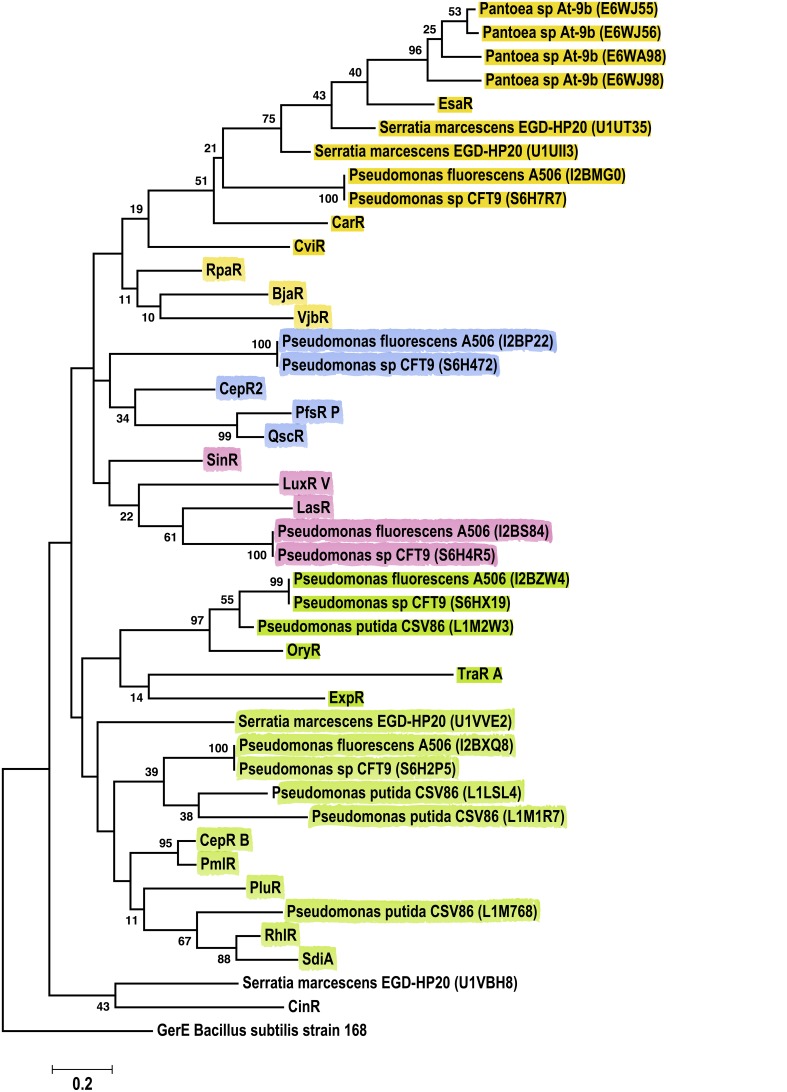
**Phylogenetic analyses of multiple LuxR solos carried by the Gammaproteobacteria**. The tree was inferred by using the Maximum Likelihood method. Tree is drawn to scale, with branch lengths measured in the number of substitutions per site. The analysis involved 44 amino acid sequences, representative from the species of the class. This included 23 Gammaproteobacterial LuxR solos represented by their Uniprot identification in the figure. All positions containing gaps and missing data were eliminated. There were a total of 64 positions in the final dataset. Colors indicate major clusters.

Phylogenetic analyses for sequences from the Gammaproteobacteria class, revealed six major clusters consistently, independent of the method (ML, NJ or Parsimony) (Figure 7). Importantly, the *Pantoea* LuxR solos included in these analyses grouped together with EsaR, CarR and CviR, whereas *Pseudomonas* sp. LuxR solos where distributed in five clusters. LuxR solos from several *Pseudomonas* sp. grouped together with OryR, TraR and ExpR; other *Pseudomonas* sp. LuxR solos grouped with EsaR and CarR suggesting relatedness to these proteins. Two *Pseudomonas* sp. LuxR solos clustered with CepR2, PfsR, and QscR whereas another two LuxR solos were found with SinR, LasR, and LuxR. The last clade of *Pseudomonas* sp. LuxR solos included characterized LuxR solo SdiA as well as QS-associated LuxRs RhlR, PmlR, CepR, and PluR. Interestingly, LuxR solos found in strains *P. fluorescens* A506, and *Pseudomonas* sp. CFT9 cluster with different characterized LuxR proteins, which indicate that each of them may have evolved from different ancestors.

These observations suggest that multiple LuxR solos carried by a bacterial species may have different evolutionary origins and likely have different ligand binding properties.

## Discussion

LuxR proteins with *N*-terminal ABD are well-studied with respect to their role in QS to regulate bacterial community behavior in proteobacteria. QS LuxRs are generally thought to bind to AHLs but recently several studies have identified novel AHL and non-AHL ligands for these proteins raising the possibility of unusual ligands and novel functions for these proteins (Schaefer et al., 2008; Ahlgren et al., 2011; Lindemann et al., 2011; Brachmann et al., 2013). In this context, studies on LuxR solos are gaining importance. Our results the analysis of sequenced genomes reveals that the majority of proteobacterial and a few non-proteobacterial genomes with QS domain LuxRs carry one or more LuxR solos. We have grouped several uncharacterized LuxR solos based on their gene context and conservation of invariant amino acids of their ABD. Several of these LuxR solos had substitutions at the invariant amino acids of the ABD raising the possibility of binding to non-AHL ligands. This approach will be of advantage to identify orthologs of LuxR solos that are otherwise difficult to compare across taxonomically distant species due to low sequence homologies.

Our results show that only 40–70% of sequenced bacterial species with QS domain LuxRs carry a complete QS system depending on taxa whereas the remaining carry only LuxR solos. In a previous study where 512 sequenced genomes were analyzed and only 26% of bacteria were reported to contain complete QS system and another 17% only QS domain LuxRs (Case et al., 2008). The differences observed in these two studies is due to the fact that our analyses is based only on genomes that contain QS domain LuxRs and does not consider other completely sequenced bacterial genomes (almost 10 times the numbers of genomes analyzed here) that lack these proteins. Overall, it is clear that among the sequenced genomes containing QS domain LuxR proteins, the majority contain LuxR solos.

Our analysis revealed the presence of genes coding for transposases and pseudogenes adjacent to the genes coding for LuxR solos. Additionally several LuxR solos also occurred near genes coding for proteins having only ABD or even another QS domain LuxR protein (Supplementary Tables 1, 2). These observations suggest a role for horizontal gene transfer and genomic rearrangement events (such as duplication or deletion) associated with occurrence of LuxR solos. LuxR solos with conserved ABD likely evolved by loss of LuxI homolog as related species carrying both *luxR*/*luxI* at the same genomic context were identified in a previous study for SdiA similar to our observations for other LuxR solos (Sabag-Daigle and Ahmer, 2012). The presence of proteins having only autoinducer domain needs to be looked into as several species harbor these proteins; although these proteins have only the ABD, some of these proteins are longer than QS domain LuxRs (Supplementary Table 2). TrlR of *A. tumefaciens* has only ABD and it is known to bind to TraR forming dimers and block its function (Chai et al., 2001). It is possible that ABD proteins function to sequester their cognate ligands or interact with QS domain LuxRs in other bacteria.

The very small numbers of LuxR solos that were detected in non-proteobacterial genomes showed only a limited level of relatedness to proteobacterial QS domain LuxR proteins. Although it is not known if these proteins are functional, it will be interesting to find out if these LuxR solos bind to AHLs or non-AHL ligands. In our analyses we also identified two QS domain LuxR proteins and a gene coding for LuxI homolog in a genetically unlinked locus of *Enterococcus gallinarum*, a Gram-positive bacterium (Supplementary Table 2). This raises the possibility that AHLs may be produced by Gram-positive bacteria as well. In fact, recently, a Gram-positive bacterium belonging to *Exuigobacterium* genera has been reported to produce 3-oxo-C8 HSLs (Biswa and Doble, 2013). Promoter regions of the non-proteobacterial LuxR solos showed the presence of 20-bp palindromic sequences (data not shown); however their role in LuxR solo mediated regulation needs to be determined.

Functionally characterized LuxR solos with known ligands are very few in number (Patankar and Gonzalez, 2009b; Subramoni and Venturi, 2009a). We have sorted LuxR solos from sequenced genomes into functionally relevant groups to understand their relatedness to well-studied LuxR solos and their probable biological roles. However, LuxR solos show only 18–25% homology to each other and it is difficult to group them into clusters based on sequence similarity as revealed in our analysis of these proteins using CLANS method. Using an alternate approach of conservation of flanking genes and invariant amino acids of ABD, we grouped a larger number of LuxR solos in to putative orthologs (Supplementary Tables 4, 5). The ortholog proteins of each group identified here are not comprehensive as it is likely that the genomic context of LuxR solo orthologs may have diverged during evolution; this might account for a number of LuxR solos that could not be grouped.

Genes coding for characterized LuxR solos are known to occur along with functionally linked genes and often regulate their expression. For example, QscR is known to regulate expression of the adjacent phenazine biosynthetic operon and XccR/OryR regulates adjacent proline imino peptidase (*pip*) (Chugani et al., 2001; Mavrodi et al., 2001; Ledgham et al., 2003; Ferluga et al., 2007; Zhang et al., 2007). Our analysis of genomic context of LuxR solos in each group revealed that several LuxR solos occur in a transcriptional unit with neighboring genes; it is possible that these genes are functionally associated with LuxR solos. The presence of 20-bp palindromic sequences were detected upstream of these transcriptional units (Supplementary Table 5); however further analyses and experimental verification will be required to confirm whether they are *lux box* motifs actually bound by LuxR solos. Interestingly, the sequence of several of these palindromic motifs appeared to be conserved within the promoters of LuxR solos of a particular group. Palindromic sequences were also detected upstream of −35 box of promoters of several Alphaproteobacterial LuxR solo containing transcriptional units of environmental/marine strains but they lacked the CT(N_12_)AG motif typical of known *lux box* sequences (Supplementary Table 5). Further analysis of promoter regions of flanking genes and experimental validation has to be carried out to understand regulation of neighboring genes by these unknown LuxR solos.

Multiple LuxR solos present in the same genome showed different levels of relatedness to characterized QS domain LuxRs suggesting different ligand binding properties and different origins. In particular, multiple LuxR solos were found in several plant-associated Alphaproteobacteria, *Pseudomonas* sp., and *Burkholderia* sp. suggesting a role for these proteins in adaptation of these bacteria to diverse habitats. It is possible that LuxR solos of different ligand specificity were acquired by these bacteria from different sources by horizontal gene transfer as it is known to be highly prevalent in many of these species, especially those belonging to *Pseudomonas* sp. (Qiu et al., 2009; Subramoni et al., 2011). This seems to be the case for the multiple LuxR solos from *Pseudomonas fluorescens* A506, which branch out with different characterized LuxR solos in the phylogenetic analyses (Figure 7); bootstrap values also support this grouping. Further analysis of the conservation of invariant amino acids of ABD and ligand binding experiments are required to understand the different roles of multiple LuxR solos in the same genome.

In summary, the systematic analysis and functional grouping of LuxR solos carried out in this study provides new information regarding the taxonomic and niche specific distribution, evolutionary origins, variation in ligand binding domain and probable roles of these proteins in bacteria which could be studied in the future.

### Conflict of interest statement

The authors declare that the research was conducted in the absence of any commercial or financial relationships that could be construed as a potential conflict of interest.
